# Expression analysis of extracellular proteins from *Phanerochaete chrysosporium* grown on different liquid and solid substrates

**DOI:** 10.1099/mic.0.2006/000513-0

**Published:** 2007-09

**Authors:** Shin Sato, Feng Liu, Hasan Koc, Ming Tien

**Affiliations:** Department of Biochemistry and Molecular Biology, Pennsylvania State University, University Park, PA 16802, USA

## Abstract

White-rot fungi secret a large number of hydrolytic and oxidative enzymes for degradation of lignocellulosic material. The sequencing of the genome of the white-rot fungus *Phanerochaete chrysosporium* has facilitated the characterization of its complete extracellular proteome. *P. chrysosporium* was grown on liquid medium, containing glucose, cellulose or wood chips as the carbon source, and also in solid substrate fermentation bags. For liquid-grown cultures, the extracellular protein fraction was separated by 2D gel electrophoresis. Protein spots were analysed by in-gel digestion and liquid chromatography (LC)/MS/MS. A total of 18 additional protein spots from the 2D gels yielded hits from blast searches. From solid substrate cultures in which the fungus was grown in bags, the proteins were resolved by SDS-PAGE, subjected to in-gel digestion and then identified by LC/MS/MS. An additional 16 proteins yielded hits on blast searches. Enzymes involved in cellulose, hemicellulose, lignin and protein degradation were identified. Expression patterns were very similar between cellulose-grown cultures and wood-grown cultures. In addition to enzymes which act on lignocellulosic material, proteases were also found, indicating the need of fungi to scavenge for nitrogen in wood.

## INTRODUCTION

Woody biomass is a complex mixture of cellulose, hemicellulose and lignin. Cellulose makes up approximately 45 % of wood by weight and hemicellulose approximately 25 % by weight. Enzymes involved in degradation of these two polymers must hydrolyse not only the glycosidic bonds that join the monomeric subunits, but also the sugars which modify the hemicellulose (Kirk & Cullen, 1998). These side groups render hemicellulose non-crystalline because they prevent efficient packing of hemicellulose. Lignin is the most complex of the three polymers and can constitute over 25 % by weight of woody biomass (Adler, 1977). Lignin is a polymer of *p*-coumaryl, coniferyl and sinapyl alcohols (van Rensburg *et al.*, 2000). The complexity of lignin arises from the 12 different types of linkages that link the monomers (Adler, 1977).

To gain access to the cellulose, microbes need to circumvent the lignin barrier (Baker, 1973). Research thus far has shown that fungi are the only microbes that can degrade lignin (Crawford & Crawford, 1980). White-rot fungi have the apparently unique ability to degrade lignin to the level of CO_2_ (Kirk & Farrell, 1987). Due to the heterogeneity of the substrate, white-rot degradation of lignocellulosic material involves an ensemble of extracellular enzymes. The carbohydrate component that links monomeric units through glycosidic bonds is readily broken by hydrolytic enzymes. Lignin, however, which contains ether bonds and carbon–carbon linkages, cannot be hydrolysed at physiological or slightly acidic pH. Its degradation involves oxidative reactions (Kirk & Farrell, 1987).

Traditional microbiological and biochemical methods have been used for over 30 years to characterize the enzymes of lignocellulose degradation. Sequencing of the *Phanerochaete chrysosporium* genome and advances in instrumentation have revolutionized methods to characterize these enzymes (Martinez *et al.*, 2004). The determination of the complete extracellular proteome is now possible. Our most recent paper describes methods to isolate the extracellular enzymes from wood-grown cultures such that they can be resolved by 2D gel electrophoresis (Abbas *et al.*, 2005). We also describe the subsequent use of liquid chromatography (LC)/MS/MS to identify 15 of the extracellular enzymes produced during growth on woody biomass. While we characterized the proteins produced with wood as the carbon source, Vanden Wymelenberg *et al*. (2005, 2006) have grown *P. chrysosporium* on cellulose and on defined media. By analysing the annotated sequences, those workers were able to predict a ‘secretosome’. Using this subset of sequences, Vanden Wymelenberg *et al.* (2005, 2006) were able to identify a large array of proteins secreted with cellulose as the growth substrate. The present paper continues our proteomic characterization of the extracellular enzymes expressed when *P. chrysosporium* is grown on wood. In addition, we characterize the extracellular proteins expressed by *P. chrysosporium* when grown on cellulose and glucose medium. We also use PCR to confirm the identity and the expression of these proteins.

## METHODS

### *P. chrysosporium* cultivation.

*P. chrysosporium* strain BKM-F-1767 (ATCC 24725) was maintained on malt agar slants, as described previously (Tien & Kirk, 1988). Fungal cultures were grown either in liquid or on solid substrates, as previously described. Solid substrate (wood) cultures were grown either in polypropylene bags in which the water content was 50 % (see below) or as ‘submerged’ cultures. The submerged cultures contained largely liquid; these liquid medium preparations were as previously described (Abbas *et al*., 2005; Tien & Kirk, 1988), containing a 1 % carbon source of glucose, cellulose or wood. Liquid stationary culture was grown at 37 ºC, as described by Abbas *et al*. (2005). The culture was flushed with water-saturated O_2_ on day 2 and every 3 days thereafter. When grown on solid substrate medium, *P. chrysosporium* inoculum was prepared by growth in 250 ml Erlenmeyer flasks containing 20 g millet, 10 g wheat bran and 30 ml water at 30 °C for 7 days, as previously described (Abbas *et al.*, 2005; Orth *et al.*, 1993). These cultures were used to inoculate polypropylene growth bags (Unicorn) containing 850 g red oak sawdust, 100 g millet, 50 g wheat bran and 1 l distilled water, and grown at 39 °C for various time periods. Proteins were extracted as described previously (Abbas *et al.*, 2005) by adding 1 vol. 0.5 M NaCl and incubating for 2 h at 4 °C with stirring. Proteins were separated from the solid substrate by filtering through cheesecloth and then centrifuged at 15 000 ***g*** for 30 min at 4 °C. Ammonium sulfate was then added to the supernatant to 100 % saturation over 30 min with constant stirring, and incubated overnight at 4 °C with stirring. The protein pellet was obtained by centrifugation at 15 000 ***g*** for 30 min at 4 °C. The pellet was dissolved in 50 ml water. Trace ammonium sulfate was removed by repeated concentration (Amicon, 10 kDa cutoff) and resuspension in 50 ml water four times.

### Protein extraction.

For liquid stationary cultures, the culture was centrifuged at 8 000 ***g***, 4 °C for 30 min. The pellet-containing mycelium was rapidly frozen in liquid N_2_ for RNA extraction. The supernatant, containing extracellular proteins, was concentrated 100-fold by an Amicon 10 kDa filter at 4 °C. To remove salt in the sample, TCA was added to a final concentration of 15 % (w/v). The sample was kept on ice for 30 min and centrifuged at 14 000 ***g*** at 4 °C for 15 min. The pellet was washed with cold acetone and then air-dried. The protein pellet was then redissolved in immobilized pH gradient (IPG) rehydration buffer (8 M urea, 2 % CHAPS, 0.4 % pH 3–10 IPG buffer, 50 mM DTT, 0.002 % bromophenol blue). Protein content was measured by Bio-Rad protein assay. Samples were stored at −70 °C.

### Assay for protease activity.

Protease activity was assayed with Azocoll as a substrate (Sigma), as described by Dosoretz *et al.* (1990). Shallow stationary cultures were assayed by incubating 1 ml extracellular fluid with 20 mg Azocoll in 50 mM acetate buffer, pH 4.5, at 37 °C. The reaction was stopped by addition of 0.4 ml 10 % TCA, followed by centrifugation at 12 000 ***g*** for 8 min at room temperature. The coloured supernatant was assayed at 520 nm against a blank sample without enzyme. One unit of enzyme activity was defined as the amount of enzyme that catalysed the release of one micromole of azo dye per minute (Dosoretz *et al.*, 1990).

### Zymogram analysis of protease.

Zymogram analysis was carried out in a 12 % polyacrylamide gel containing 0.05 % casein as the protein substrate (Wilson & Howlett, 2005). The concentrated extracellular fluid, as described above, was incubated in 0.02 M Tris/HCl (pH 8.0) at room temperature for 20 min. SDS-PAGE sample buffer was then added to the enzyme solutions. After incubation at 42 °C for 1 h, 40 μl was applied to the casein-containing SDS-PAGE gel. The gel was run at 15 mA for 1.5 h at 4 °C. After electrophoresis, the gel was incubated in 2.5 % Triton X-100 for 30 min at room temperature, followed by 0.05 M Tris/HCl, 200 mM NaCl, 6 mM CaCl_2_, pH 7.5, for 16 h at room temperature. The gel was stained with Coomassie brilliant blue R250.

### Xylanase assay.

Xylanase activity was measured as described by Bailey *et al.* (1992). Xylan from birch wood (Sigma) was used as a substrate, and was used as a 1.25 % (w/v) solution in 50 mM citrate buffer (pH 5.3), as described by Bailey *et al.* (1992). Reaction mixtures of 1.25 ml contained 50 μg extracellular protein and 1 % xylan in 50 mM citrate buffer (pH 5.3). Shaking reaction mixtures were incubated at 37 °C and sampled at 0, 30, 60 and 90 min by removing 100 μl and adding to 100 μl dinitrosalicylic acid (Miller, 1959). After boiling for 5 min, 34 μl 40 % sodium potassium tartrate solution was added. After cooling, 100 μl reaction mixture was added to 900 μl water and *A*_575_ was measured. Three replicates of the same sample were analysed. Commercial xylose (Sigma) was used as a standard. One unit of xylanase activity was defined as the amount of enzyme that catalysed the release of one micromole of xylose per minute.

### Electrophoresis.

Protein samples were subjected to SDS-PAGE (Laemmli, 1970) and also to 2D gel electrophoresis. For 2D gels, extracellular protein samples (∼100 μg) from different carbon source cultures, wood, glucose or cellulose, were used to rehydrate IPG strips, 11 cm, pH 3–10 (Amersham Biosciences). Protein samples (∼300 μg) from 6, 12, 18 and 30 day wood-grown cultures were used to rehydrate IPG strips, 11 cm, pH 3–7.3 (basic end-cut 18 cm strips) (Amersham Biosciences). The first-dimensional IEF was run at 8000 V (current limit at 50 μA per gel) using a Protean IEF cell (Bio-Rad) for a total of 30 000 V h. IPG strips were equilibrated for 10 min in reducing buffer (50 mM Tris/HCl, pH 6.8, 6 M urea, 30 %, v/v, glycerol, 2 % SDS, 0.002 % bromophenol blue, 10 mg DTT ml^−1^), and then 10 min in alkylating buffer (50 mM Tris/HCl, pH 6.8, 6 M urea, 30 %, v/v, glycerol, 2 % SDS, 0.002 % bromophenol blue, 25 mg iodoacetamide ml^−1^). The IPG strips were placed on top of 12 % SDS-PAGE gels and run on a Hoefer SE 600 cooled vertical electrophoresis unit 18×16 cm at 300 V until the bromophenol blue reached the bottom of the gel. Gels were stained with Coomassie brilliant blue R-250.

### In-gel digestion and LC/MS/MS.

Protein spots from SDS-PAGE and from 2D gels were excised with a clean razor blade and digested by trypsin, as described previously (Abbas *et al.*, 2005), with the following modifications: the extracted peptides were dissolved in 10 μl double-distilled H_2_O instead of trifluoroacetic acid, and no desalting step was performed. Peptide identification by LC/MS/MS was the same as that of Abbas *et al.* (2005).

### RNA extraction.

The mycelium precipitates from 6, 12, 18 and 30 day wood-grown cultures were ground into fine powder in the presence of liquid N_2_. Total RNA was extracted using TRIZOL reagent (Invitrogen). RNase-free DNase (Promega) was added to the RNA solution at 1 U (μg RNA)^−1^ and incubated at 37 °C for 30 min to remove genomic DNA. After inactivating DNase at 65 °C for 10 min, samples were used immediately to perform the reverse transcription.

### Relative quantitative RT-PCR (RQ RT-PCR).

Temporal gene expression of extracellular proteins was analysed by RQ RT-PCR. Approximately 2 μg RNA from each time point was used in a reverse transcription reaction primed with 1 μg random primers (Promega) at 37 °C for 1 h. PCR reactions were performed with the Eppendorf Mastercycler gradient. RQ RT-PCR followed the procedure described in the QuantumRNA 18S Internal Standards kit (Ambion). Briefly, PCR reactions contained two sets of primers, primers for the gene of interest and for an internal standard. The quantity of target gene was expressed as a ratio of this gene to that of the internal standard, in this case 18S rRNA (rRNA). The cycle number of PCR must be within the linear range (cycles vs logarithm of product) of the target gene, which was determined by 10 identical PCR reactions, except that the number of cycles was increased by two. In order to obtain reliable data, the levels of the internal standard and target gene should be similar. Since rRNA is very abundant, competimers (Ambion) were added to the primer mixture at different ratios. A total of nine genes (primer sequences in Table 1) from previously identified extracellular enzymes (Abbas *et al.*, 2005) were analysed by RQ RT-PCR. All PCR reactions were performed in a 20 μl reaction volume and under the following conditions: initial denaturation of 3 min at 95 °C, followed by amplification cycles (the cycle number was chosen from the linear range of the PCR reaction and was distinctive for each gene) of 95 °C for 30 s, 55 °C for 30 s, 72 °C for 30 s, and a final extension of 10 min at 72 °C (this step was eliminated in PCRs for linear range determination). For PCR reactions containing only one pair of primers, the component amounts were 0.5 U GoTaq DNA polymerase (Promega), 1× PCR buffer, 250 μM each dNTP, 0.5 μM each primer, and 1 μl cDNA template. For duplex PCR reactions (containing a certain ratio of 18S rRNA primers and competimers to gene-specific primer pair), the component amounts were 2.5 U GoTaq DNA polymerase (Promega), 1.6× PCR buffer, 500 μM each dNTP, 0.5 μM each primer, and 1 μl cDNA template. PCR products were separated on 1.4 % agarose gels, visualized with ethidium bromide, quantified by Scion Image (Scion) and plotted in Excel. The PCR products of target genes were further verified by DNA sequencing (Nucleic Acid Facility, Pennsylvania State University).

## RESULTS

### Protein identification from wood cultures

**Fig. 1. f1:**
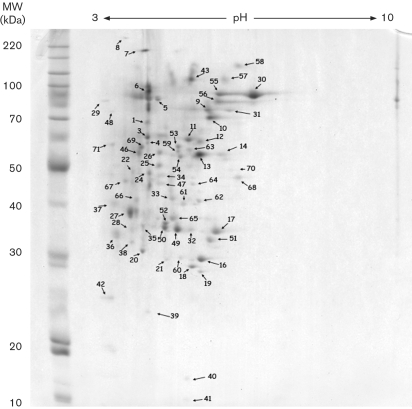
2D gel of extracellular proteins from *P. chrysosporium* grown on submerged liquid cultures with red oak sawdust as the carbon source. MW, molecular weight.

In our previous study (Abbas *et al.*, 2005), *P. chrysosporium* was grown using wood as the carbon source in submerged, shallow stationary cultures. Using 2D gels with in-gel digestion and LC/MS/MS, we previously identified 16 proteins from the extracellular proteome of *P. chrysosporium*. In the present paper, *P. chrysosporium* was again grown on submerged wood medium with the objective of determining the extracellular proteome (Fig. 1). A total of 41 protein spots were excised from the gel, digested in-gel with trypsin and analysed by LC/MS/MS. From these 41 spots, we were able to obtain blast hits to 34 proteins using the annotated *P. chrysosporium* database (http://genome.jgi-psf.org/whiterot1/whiterot1.home.html) (Table 2). Some of the spots yielded identical blast hits, suggesting the presence of different isoforms of the extracellular enzymes.

### Proteins expressed in solid substrate cultures

In our previous paper (Abbas *et al.*, 2005), we described problems associated with a brown-coloured, probably lignin-derived extract. This extract caused streaking on 2D gel electrophoresis. The extensive streaking from the extract caused difficulty in the visualization and subsequent digestion of proteins. For the purpose of identifying the complete proteome, the protein extract was not resolved by 2D gels. Total extracellular protein extracts were digested with trypsin in solution and then analysed by LC/MS/MS. The extracellular extracts, however, were not easily digested in solution. Large protein bands were still visible on SDS-PAGE, even after overnight digestion. This could be due either to the presence of protease inhibitors in the wood extract or to the fact that the extracellular proteins, without prior denaturation with SDS, were highly resistant to protease digestion. Based on the findings described below, we favour the latter possibility. The proteins were then separated by SDS-PAGE. The proteins from the whole SDS-PAGE lane were then subjected to in-gel digestion, and the total peptide mixture was analysed by LC/MS/MS. The results are shown in Table 3. This analysis resulted in an additional eight proteins yielding positive blast hits. Enzymes involved in cellulose degradation and proteases were detected in this mixture. Oxalate decarboxylase was also detected.

### Protein expression from glucose and cellulose liquid cultures

**Fig. 2. f2:**
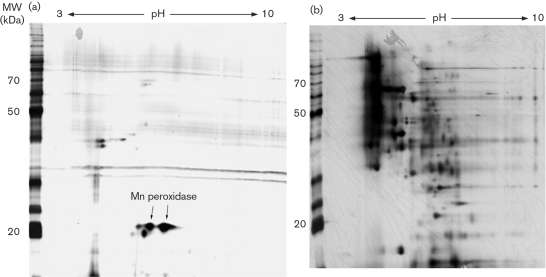
2D gels of extracellular proteins from *P. chrysosporium* grown on carbon sources of (a) glucose and (b) cellulose. MW, molecular weight.

To determine the profile of enzymes uniquely expressed for growth on wood, *P. chrysosporium* was also grown in nitrogen-limited glucose and cellulose cultures. Cellulose cultures are also relevant, since Vanden Wymelenberg *et al.* (2005) have characterized the extracellular proteome of cellulose-grown cultures. The 2D gels of extracellular proteins from glucose- and cellulose-grown cultures are shown in Fig. 2. *P. chrysosporium* grown in glucose culture produced the fewest proteins. As indicated on the gel, we were able to detect Mn peroxidase in these liquid cultures (running anomalously low). Analysis of the gels from wood-containing media showed no spots in the same region of the gel.

The cellulose-grown culture yielded protein patterns very similar to those of wood-grown cultures (Fig. 2). However, the 2D gels showed some differences in protein spot intensities. Xylanases were not easy to detect upon visual inspection of the 2D gels from cellulose-grown cultures. We thus analysed the wood- and cellulose-grown cultures for xylanase activity, and indeed found much lower activity in cellulose-grown cultures. Whereas cellulose-grown cultures contained 0.06 U (μg protein)^ −1^, solid substrate cultures grown on red oak contained 0.38 U (μg protein)^ −1^.

### Protease activity and zymogram

**Fig. 3. f3:**
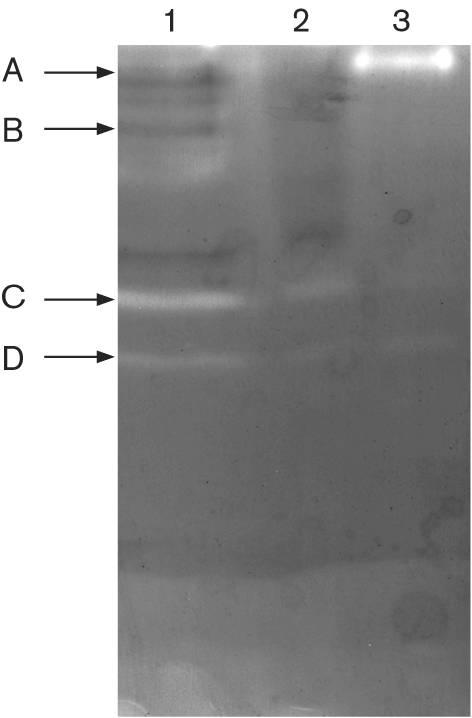
Zymogram of extracellular proteases from *P. chrysosporium*. Lanes 1, 2, and 3 contain concentrated extracellular fluid from wood-, cellulose- and glucose-grown cultures, respectively, as described in Methods. Four protease bands were detected, labelled A, B, C and D (see text).

The detection of numerous proteases in the wood-degrading cultures led to further characterization of these enzymes. Extracellular acid protease activity from five replicates of 6-day-old cultures were measured in the extracellular fluid from glucose-containing, cellulose submerged and wood-submerged culture of *P. chrysosporium*. The wood-submerged culture contained the highest protease activity (3.05±0.15 U l^−1^). Slightly lower protease activity was detected in the cellulose-grown cultures (2.25±1.06 U l^−1^). Glucose-containing culture contained very little protease activity (0.86±0.36 U l^−1^). To determine whether the cultures produced the same proteases, we performed native gel electrophoresis and zymograms. In wood-grown cultures, at least four different proteases could be detected (Fig. 3). Cellulose-grown cultures produced at least two proteases, both of which are found in wood-grown cultures. In glucose-grown cultures, three different proteases could be observed, with the predominant enzyme having a higher pI (band A). In the glucose-containing and cellulose-submerged culture, two protease isozymes were detected on the gel. At least three protease isozymes were found in the wood-submerged culture.

### Temporal expression analysis of extracellular proteins

**Fig. 4. f4:**
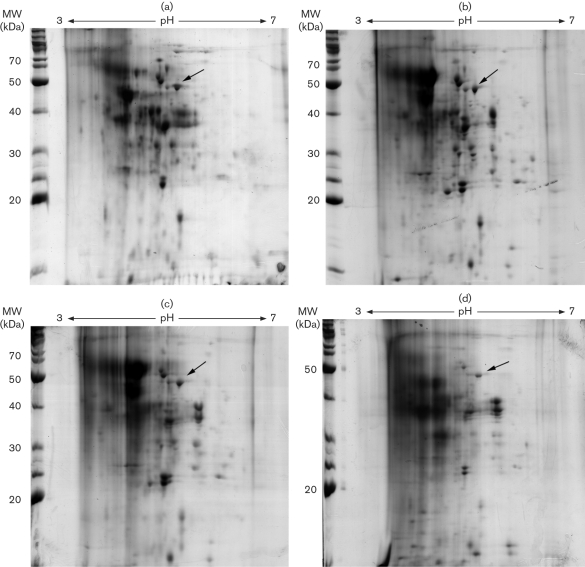
2D gels of temporal expression of extracellular proteins from *P. chrysosporium* grown on sawdust as carbon source. (a) 6 day culture; (b) 12 day culture; (c) 18 day culture; (d) 30 day culture. Arrow shows endopolygalacturonase as identified by LC/MS/MS. MW, molecular weight.

To determine whether the suite of enzymes produced by *P. chrysosporium* changed over time, extracellular proteins were obtained at 6, 12, 18 and 30 days from *P. chrysosporium* cultured on liquid wood medium (Fig. 4). 2D gels were performed on triplicate samples. We found that protein preparations from samples of different ages also contained different amounts of the brown extract (data not shown), making integration of spots more difficult (thus, representative gels are shown in Fig. 4). Analyses of triplicate gels, a standard for proteomic analysis, was not feasible with wood-grown cultures. However, close examination of the representative gels could be generalized by stating that most, but not all, protein spots changed in intensity. For example, the arrow in Fig. 4 points to endopolygalacturonase, previously identified by LC/MS/MS (Abbas *et al.*, 2005). The pattern shows approximately equal levels (if not a slight increase) at 6, 12 and 18 days, followed by a decrease at day 30.

### PCR corroboration of selected proteins

**Fig. 5. f5:**
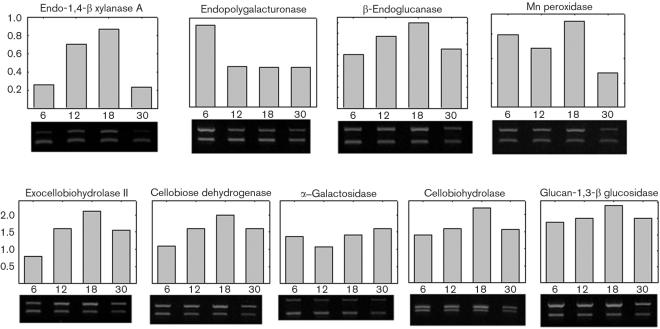
Temporal gene expression analysis by RQ RT-PCR.

To corroborate the proteomic results, RT-PCR was performed for selected proteins identified from the 2D gels. The PCR was also performed with proteins from 6, 12, 18 and 30 days. This provided information on temporal expression which could not be obtained from the 2D gels. Total RNA was extracted from the wood-grown cultures at various times, and the mRNA level of transcripts encoding selected extracellular proteins was quantified by RT-PCR. A total of nine genes were analysed. Genes encoding endopolygalacturonase, exocellobiohydrolase II, cellobiose dehydrogenase, *β*-galactosidase, cellobiohydrolase, glucan-1,3-*β* glucosidase, endo-1,4-*β* xylanase A, *β*-endoglucanase and Mn peroxidase were analysed (Table 1). The linear range for the PCR reaction was determined (data not shown). The amplification cycles are listed in Table 1. The ratio of the 18S rRNA primers to the competimers is also provided in Table 1. Gels showing the PCR products are shown in Fig. 5: the top gel band is the gene of interest and the bottom gel band is the rRNA amplification product (315 bp). The band for Mn peroxidase, a protein not detected in the 2D gels for wood-grown cultures (Fig. 2), was clearly detected by PCR. The gel images were analysed by Scion Image and the results are shown in Fig. 5. Expression levels for most of the genes increased from 6 to 18 days, and then decreased thereafter. Of the nine genes examined, only endopolygalacturonase reached a maximum level at day 6 and then decreased thereafter. Interestingly, this pattern of increase followed by decrease was observed on the protein gels (Fig. 4).

## DISCUSSION

In the present paper, we were able to obtain hits to 34 proteins from 2D gels of solid substrate submerged cultures and an additional eight proteins from SDS-PAGE gels of solid substrate cultures. We previously identified 16 protein spots on the 2D gels (Abbas *et al.*, 2005). The proteins detected can be categorized into those involved in (i) carbohydrate metabolism, (ii) lignin metabolism and (iii) protein metabolism. The identification of these proteins by LC/MS/MS was confirmed by RT-PCR of selected proteins.

In addition to our laboratory, that of D. Cullen at the University of Wisconsin, Madison, has also worked on the proteome of *P. chrysosporium*. Those workers (Vanden Wymelenberg *et al.*, 2006) analysed the *P. chrysosporium* genome of 10 048 predicted protein models with phobius (http://phobius.cgb.ki.se/index.html) predictive software for secreted proteins. This analysis resulted in a total of 769 proteins being identified as composing the secretosome (7.6 % of the *P. chrysosporium* gene models). They indicated that this list also includes cell wall-bound and endoplasmic reticulum (ER)-related proteins. Similar to the present study, those workers also analysed the extracellular proteome of *P. chrysosporium* using electrophoretic separation and LC/MS/MS. They found mannose-6-phosphatase, proteases and 28 gene products, including representatives of the following gene families: four lignin peroxidases, three lipases, two carboxylesterases and eight glycosyl hydrolases.

The culture conditions used by Vanden Wymelenberg *et al*. (2006) are different from those of our studies. In their most recent study, glucose-grown liquid shallow stationary cultures were used that were either carbon-starved or nitrogen-starved. In an earlier study, Vanden Wymelenberg *et al*. (2005) used cellulose as the carbon source and again used LC/MS/MS to analyse the extracellular proteome. Our studies have mainly focused on wood-grown cultures. While this strategy may provide a more physiologically relevant proteome, it has a large number of technical difficulties. However, as described below, both studies have found highly similar protein profiles, suggesting that the liquid glucose- and cellulose-grown cultures are viable models to study lignocellulose degradation.

### Glyco-hydrolases

Within the family of carbohydrate-metabolizing hydrolases, enzymes involved in both cellulose and hemicellulose metabolism were detected in our study. Comparison of expression patterns between cellulose- and wood-grown cultures showed very little difference. The patterns were similar, although the intensity of some protein bands was different. This suggests that the proteins characterized in the present study are similar to those found by Vanden Wymelenberg *et al*. (2005, 2006). Indeed, many of the proteins found are similar, although not identical. While our results and those of Vanden Wymelenberg *et al*. (2005) show xylanases to be produced in cellulose-grown culture, we show that xylanases are more highly expressed in wood-grown than in cellulose-grown cultures. This is consistent with earlier work on cellulose and xylanase regulation in *P. chrysosporium* (Dobozi *et al.*, 1992). Those workers showed that *P. chrysosporium* is different from most other fungi, in which the cellulases and xylanases are coordinately regulated (Han *et al.*, 2004).

Similar to the findings of Vanden Wymelenberg *et al*. (2006), no cellulases or hemicellulases were detected in liquid high-glucose, low-nitrogen cultures. This is consistent with glucose repressing the synthesis of the cellulolytic enzymes (Yoshida *et al.*, 2004).

### Peroxidases

The proteins from high-glucose, nitrogen-limited cultures have been previously characterized as mainly lignin and Mn peroxidases (Farrell *et al.*, 1989; Tien, 1987), and this was also found by Vanden Wymelenberg *et al*. (2006).

While the peroxidases were easy to find in glucose-grown cultures, they were difficult to detect by 2D gels in wood-grown cultures. Although our previous studies using Western blots have also shown that lignin peroxidase is difficult to detect in wood cultures (Orth *et al.*, 1993), we were able to detect this enzyme on the 2D gels. We were not able to detect Mn peroxidase on the 2D gels. However, previously we were able to detect Mn peroxidase activity in solid substrate cultures (Orth *et al.*, 1993), and in the present study, RT-PCR showed that Mn peroxidase was being expressed in wood cultures.

### Pectinase and oxalate decarboxylase

Pectinases are not well characterized in wood-degrading fungi. The enzyme hydrolyses pectin, which is predominantly a linear polymer of 1,4-d-galacturonic acid units and their methyl esters, interrupted in places by 1,2-linked l-rhamnose units. Green *et al.* (1993) have suggested that despite the low pectin content of wood (4 %), this enzyme activity may be important in decay because most of the pectin is located in the central region of the pit membranes, ray cell walls, and the compound middle lamellae of the wood cell wall. Also important is that the primary location of calcium in wood is in the pectin (Bednarska *et al.*, 2005). Calcium is suggested to play a central role in cell wall integrity (Jellison *et al.*, 1997; Young & Guinn, 1966).

The role of oxalate decarboxylase is not as clear as that of the other enzymes detected in the extracellular matrix. Oxalate decarboxylase has been detected (and purified) from other white-rot fungi (Dutton *et al.*, 1994). Oxalate has been postulated to facilitate cell wall degradation by complexing with calcium, thereby weakening its structure. We have found that oxalate-chelated Mn^2+^ is much more readily oxidized by Mn peroxidase than free Mn^2+^ (Kuan *et al.*, 1993). An earlier study did not detect any oxalate decarboxylase in *P. chrysosporium* (Makela *et al.*, 2002). However, those workers only examined intracellular oxalate decarboxylase, not the extracellular enzyme. They suggest that this enzyme is only involved in the regulation of oxalate levels. The *P. chrysosporium* oxalate decarboxylase is predicted to contain a 28 aa signal peptide. Thus, the enzyme does seem to be targeted to the extracellular matrix. Indeed, with brown-rot fungi, oxalate decarboxylase is located extracellularly (Micales, 1997). There are no known roles for the products of oxalate decarboxylase (formate or carbon dioxide) in wood degradation.

### Proteases/nitrogen metabolism

Another set of enzymes not involved directly with lignocellulose degradation are the proteases. Vanden Wymelenberg *et al*. (2006) found proteases in cellulose- and glucose-grown (nitrogen-limited) cultures. They found five peptidases that are members of a large family of aspartyl proteases. They also found that many of the genes are localized to gene clusters. This again suggests that expression patterns in cellulose and wood are similar. While our results are largely in agreement, we do show minor differences in protein expression. Our protease zymograms show that four proteases are produced in wood-grown cultures, and that at least three of these proteases are also produced in cellulose-grown cultures.

Despite the production of these proteases, the fungal enzymes are relatively resistant. Incubation up to 6 h did not result in appreciable degradation of extracellular fungal proteins. Similar results have been obtained in whole-culture studies in which we have shown the stability of extracellular fungal proteins for over 1 month (Pease & Tien, 1992).

In addition to aspartic protease and acid proteinase, we also found glutaminase. The detection of proteases (and other enzymes involved in nitrogen metabolism) has been previously reported (Cancel *et al.*, 1993; Dass *et al.*, 1995; Dosoretz *et al.*, 1990). However, these enzymes have not been widely studied in wood degradation and have not been thought to be of great significance. Because most laboratory studies with *P. chrysosporium* are under nitrogen-starved conditions, researchers have largely uncoupled the production of proteases from lignin degradation. This is despite the finding that nitrogen is the most limiting component for growth on wood (Van Soest, 1994). Also of relevance is that nutrient nitrogen limitation triggers the synthesis of the lignin-degrading system (Keyser *et al.*, 1978). Investigators have mostly suggested that carbon starvation is more physiologically relevant (Kirk & Farrell, 1987). However, we suggest that the fungus is bathing in carbon when growing on woody biomass. We propose that the production of proteases is part of the ligninolytic (or wood-degrading) response. This is consistent with fungi degrading lignin to gain access not just to the cellulose, but also to the nitrogen trapped in wood. Indeed, the nitrogen content of wood varies from 1 to 2 % (Van Soest, 1994), and it has been suggested that most of this nitrogen is in the form of protein.

Nitrogen acquisition from lignin-linked proteins is also consistent with our detection of glutaminase in fungal cultures. Release of ammonia from cross-linked proteins would also yield nutrient nitrogen for wood-degrading fungi. Glutaminase deaminates glutamine to yield glutamate, and has been found in other fungi (Yano *et al.*, 1991a, b).

In summary, our study has shown the large number of enzymes involved in fungal lignocellulose metabolism. We have identified a large number of proteins on 2D gels, such that future workers can use this as a database for comparison and identification of proteins. The identity of some selected proteins identified by proteomics was corroborated by RT-PCR quantification of message levels. The RT-PCR results are similar to those obtained in protein expression pattern gels. We have also shown that expression patterns are relatively similar between cellulose- and wood-grown cultures. Finally, our work has further characterized the proteases produced by *P. chrysosporium* and proposed a more significant role for these enzymes in wood degradation.

## Figures and Tables

**Table 1. t1:** Primers for PCR of *P. chrysosporium* genes

Gene*	**Forward primer sequence†**		**Ratio||**
1		35	1.3 : 8.7
2		25	2.2 : 7.8
3		30	1.5 : 8.5
4		37	1 : 9
5		27	2.2 : 7.8
6		30	1.5 : 8.5
7		33	1.5 : 8.5
8		31	1 : 9
9		28	1.7 : 8.3

*P. chrysosporium genes (1–9) encoding endopolygalacturonase, exocellobiohydrolase II, cellobiose dehydrogenase, *α*-galactosidase, cellobiohydrolase, glucan-1,3-*β* glucosidase, endo-1,4-*β* xylanase A, *β*-endoglucanase and Mn peroxidase, respectively. †Primer sequences are based on sequences from the annotated *P. chrysosporium* database. ‡Predicted size of PCR product. §The amplification cycle number was chosen from the linear PCR range for the gene. ||Ratio of 18S rRNA primers and competimers in a duplex PCR reaction.

**Table 2. t2:** Identity of protein spots shown in Fig. 1

**Spot**	**Sequence**	**Putative enzyme**	**Accession no.**	**Mass (Da)**	***E* value**
9	DPAVEYIVAGGK	Glutaminase A	Genewise2nd.38.23.1	71 862	39
	QAFGATEITASR				0.0013
	ISADYASLVALSVR				4.9e-006
	AQFVNSGTLPNTQDTR				0.0023
	QSLPEFYVTLTATSTDGK				6.7e-006
20	LQAATQWLQQNNLK	Endoglucanase	Genewise.6.4.1	41 354	0.0014
24	ISADYASLVALSVR	Glutaminase A	Genewise2nd.38.23.1	71 862	5.3e-006
25	ATSLLNLVK	Endo-1,4-*β* xylanase A	Genewise2nd.42.28.1	42 594	21
	LYINEYNIEFAGAK				2.5e-006
26	ATSLLNLVK	Endo-1,4-*β* xylanase A	Genewise2nd.42.28.1	42 594	1.4
	LYINEYNIEFAGAK				0.00051
31	DPAVEYIVAGGK	Glutaminase A	Genewise2nd.38.23.1	71 862	0.022
	ISADYASLVALSVR				4.2e-006
	FWTGSILGWAGFIK				0.59
	AQFVNSGTLPNTQDTR				0.71
34	LYINEYNIEFAGAK	Endo-1,4-*β* xylanase A	Genewise2nd.42.28.1	42 594	0.00047
35	GTLTSDGATYDVYEGTR	Endo-1,4-*β* xylanase B	pc.120.11.1	30 804	0.0036
43	ATSLLNLVK	Endo-1,4-*β* xylanase A	Genewise.74.21.1	44 057	12
	MTLPSTPALLAQQK				0.86
	LYINEYNIEYAGAK				0.045
	FTAAQLTSIIQNHCSTLVTHYK				0.26
52	VGFAPVVLK	Aspartic protease precursor	pc.163.11.1	35 031	1.9
	TFTTEFADGSTVQGEVFK				0.0056
53	IVLDANWR	Exocellobiohydrolase I precursor	pc.139.26.1	56 218	1.7
	LQFVTGSNVGSR				8.3e-006
	TAFGDTNYFAQHGGLK				0.13
	DTGLCDADGCDFNSFR				0.00028
57	ATSLLNLVK	Endo-1,4-*β* xylanase A	Genewise.74.21.1	44 057	3.5
	KPAYDGIAIGFGN				41
	MTLPSTPALLAQQK				0.015
	LYINEYNIEYAGAK				5.6e-005
	FTAAQLTSIIQNHCSTLVTHYK				4.6
58	LYNLDIR	Pectin methylesterase	Genewise2nd.56.10.1	33 613	0.0083
	EGSAYFAGNTIATK				0.012
59	IVLDANWR	Exocellobiohydrolase I precursor	pc.139.26.1	56 218	0.81
	LQFVTGSNVGSR				0.14
	DTGLCDADGCDFNSFR				7.5e-005
60	GTLTSDGATYDVYEGTR	Endo-1,4-*β* xylanase B	pc.120.11.1	30 804	0.0054
65	GTLTSDGATYDVYEGTR	Endo-1,4-*β* xylanase B	pc.120.11.1	30 804	0.00045
66	GTLTSDGATYDVYEGTR	Endo-1,4-*β* xylanase B	pc.120.11.1	30 804	0.049
68	FAISNWGVDPNR	Acetyl xylan esterase	pc.100.54.1	35 963	9.6e-006

**Table 3. t3:** Results of blast homology search of proteins from SDS-PAGE gel

**Sequence**	**Putative enzyme**	**Accession no.**	**Mass (Da)**	***E* value**
TFQTGSSSTAVDQR	Aspartic protease	pc.163.11.1	35 031	0.58
TFTTEFADGSTVQGEVFK				3.8e-006
TFQTGSSSTAVDQR				0.25
NLYTEFDFGNLR				0.00033
TFTTEFADGSTVQGEVFK				7.3
VSAGVFAFK	Aspartic protease	pc.127.30.1	43 951	2.1
LASSGSTLFLGGTDTSK				1.8e-011
VSAGVFAFK				0.26
LASSGSTLFLGGTDTSK				1.2e-008
TGSGSVGPSGANIIDIQQ	Acid proteinase EapC precursor	pc.123.27.1, pc.123.29.1	26 643	6.6e-007
VNYVDQATALAK	Endo-1,3(4)-*β* glucanase	pc.78.37.1	34 396	0.36
NLTYASGDTLILR				0.03
IMNQDDCLAINK	Endopolygalacturonase	Genewise2nd.1.81.1	32 826	0.017
GTLTSDGATYDVYEGTR	Endo-1,4-*β* xylanase B	pc.120.11.1	30 804	4e-009
GTLTSDGATYDVYEGTR				8.3e-009
QLVQIVIYDLPDR	Exocellobiohydrolase CBHII	pc.3.82.1	48 957	0.007
IPDLGTYLASASALGK				0.15
AASVANIPTFTWLDSVAK				0.19
QLVQIVIYDLPDR				6.7e-005
IPDLGTYLASASALGK				5.6e-011
AASVANIPTFTWLDSVAK				0.74
VVAVIEPDSLANLVTNLN				0.0063
NTGLCDGDGCDFNSFR	Exocellobiohydrolase I precursor	pc.33.51.1	59 474	1.7e-006
NTGLCDGDGCDFNSFR				1.2e-006
SNNPNGFADTITAWTR	Exo-1,3-*β* glucanase	pc.67.67.1	83 143	7.4e-007
GDGNTDDTAAIQAAINAG				7.1e-010
VSSPLVVLYQTQLIGDAK				6.9e-006
NFVIDLR				0.012
NPDTGAQFIIVR	*β*-Galactosidase	pc.96.2.1	104 589	4.1e-005
LPVPDLWLDIFQK				3.8e-005
NPDTGAQFIIVR				9.8e-005
VILTDYTFGNPANANK				0.11
VNYVNQATAVAK	Glucanase precursor	pc.22.125.1	34 352	2.3
ADDTTVLSPSGPGR				0.0082
FPGLCLEDSPLGVR	*β*-Glucosidase	pc.2.181.1	87 055	0.00033
GANIYGLGEVVASSGFR	*α*-Glycosidase precursor	pc.156.9.1	104 023	0.0012
APSYVFSVLNDIK	Glycosyl hydrolase family 30	pc.202.4.1	47 509	0.0033
LSGMPATQLQGGSVK	Oxalate decarboxylase	pc.137.15.1	52 097	0.0074
LSGMPATQLQGGSVK				0.014
VLGIGYVQELVAR		Genewise2nd.32.11.1	23 301	1.6e-007
FCGLFTEEEWR				4.5e-005
VLGIGYVQELVAR				5.9e-007
FCGLFTEEEWR				0.1
ATGATLDNNTGLLR	Polyporopepsin (aspartic proteinase)	pc.12.138.1	45 212	2.1e-007
FTGSITYTSLTK				0.0016
ATGATLDNNTGLLR				1.4e-006
GASQWAFQLDGVISR	Not known	Genewise2nd.32.31.1	45 973	0.012
SGCSGDCNQLSSCGTR	Not known	pc.124.7.1	15 331	0.00069

## Abbreviations

- IPG, immobilized pH gradient
- LC, liquid chromatography
- RQ RT-PCR, relative quantitative RT-PCR

## References

[r1] Abbas, A., Koc, H., Liu, F. & Tien, M. (2005). Fungal degradation of wood: initial proteomic analysis of extracellular proteins of *Phanerochaete chrysosporium* grown on oak substrate. Curr Genet 47, 49–56. 15551134 10.1007/s00294-004-0550-4

[r2] Adler, E. (1977). Lignin chemistry – past, present and future. Wood Sci Technol 11, 169–218.

[r3] Bailey, M. J., Biely, P. & Poutanen, K. (1992). Interlaboratory testing of methods for assay of xylanase activity. J Biotechnol 23, 257–270.

[r4] Baker, A. J. (1973). Effect of lignin on the in vitro digestibility of wood pulp. J Anim Sci 35, 768–771.

[r5] Bednarska, E., Lenartowska, M. & Niekras, L. (2005). Localization of pectins and Ca^2+^ ions in unpollinated and pollinated wet (*Petunia hybrida* Hort.) and dry (*Haemanthus albiflos* L.) stigma. Folia Histochem Cytobiol 43, 249–259. 16382894

[r6] Cancel, A. M., Orth, A. B. & Tien, M. (1993). Lignin and veratryl alcohol are not inducers of the ligninolytic system of *Phanerochaete chrysosporium*. Appl Environ Microbiol 59, 2909–2913. 8215363 10.1128/aem.59.9.2909-2913.1993 PMC182385

[r7] Crawford, D. L. & Crawford, R. L. (1980). Microbial degradation of lignin. Enzyme Microb Technol 2, 11–22.

[r8] Dass, S. B., Dosoretz, C. G., Reddy, C. A. & Grethlein, H. E. (1995). Extracellular proteases produced by the wood-degrading fungus *Phanerochaete chrysosporium* under ligninolytic and non-ligninolytic conditions. Arch Microbiol 163, 254–258. 7763133 10.1007/BF00393377

[r9] Dobozi, M. S., Szakacs, G. & Bruschi, C. V. (1992). Xylanase activity of *Phanerochaete chrysosporium*. Appl Environ Microbiol 58, 3466–3471. 16348798 10.1128/aem.58.11.3466-3471.1992 PMC183130

[r10] Dosoretz, C. G., Chen, H. C. & Grethlein, H. E. (1990). Effect of environmental conditions on extracellular protease activity in lignolytic cultures of *Phanerochaete chrysosporium*. Appl Environ Microbiol 56, 395–400. 16348114 10.1128/aem.56.2.395-400.1990 PMC183351

[r11] Dutton, M. V., Kathiara, M., Gallagher, I. M. & Evans, C. S. (1994). Purification and characterization of oxalate decarboxylase from *Coriolus versicolor*. FEMS Microbiol Lett 116, 321–325.

[r12] Farrell, R. L., Murtagh, K. E., Tien, M., Mozuch, M. D. & Kirk, T. K. (1989). Physical and enzymatic properties of lignin peroxidase isoenzymes from *Phanerochaete chrysosporium*. Enzyme Microb Technol 11, 322–328.

[r13] Green, F., III, Kuster, T. A. & Highley, T. L. (1996). Pectin degradation during colonization of wood by brown-rot fungi. Recent Research Developments in Plant Pathology 1, 83–93.

[r14] Han, S. O., Cho, H. Y., Yukawa, H., Inui, M. & Doi, R. H. (2004). Regulation of expression of cellulosomes and noncellulosomal (hemi)cellulolytic enzymes in *Clostridium cellulovorans* during growth on different carbon sources. J Bacteriol 186, 4218–4227. 15205424 10.1128/JB.186.13.4218-4227.2004 PMC421611

[r15] Jellison, J., Connolly, J., Goodell, B., Doyle, B., Illman, B., Fekete, F. & Ostrofsky, A. (1997). The role of cations in the biodegradation of wood by the brown rot fungi. Int Biodeterior Biodegrad 39, 165–179.

[r16] Keyser, P., Kirk, T. K. & Zeikus, J. G. (1978). Ligninolytic enzyme system of *Phanaerochaete chrysosporium*: synthesized in the absence of lignin in response to nitrogen starvation. J Bacteriol 135, 790–797. 690075 10.1128/jb.135.3.790-797.1978 PMC222449

[r17] Kirk, T. K. & Cullen, D. (1998). *Enzymology and Molecular Genetics of Wood Degradation by White-Rot Fungi. Environmentally Friendly Technologies for the Pulp and Paper lndustry*. Hoboken, NJ: John Wiley & Sons.

[r18] Kirk, T. K. & Farrell, R. L. (1987). Enzymatic combustion: the microbial degradation of lignin. Annu Rev Microbiol 41, 465–505. 3318677 10.1146/annurev.mi.41.100187.002341

[r19] Kuan, I. C., Johnson, K. A. & Tien, M. (1993). Kinetic analysis of manganese peroxidase: the reaction with manganese complexes. J Biol Chem 268, 20064–20070. 8376363

[r20] Laemmli, U. K. (1970). Cleavage of structural proteins during the assembly of the head of bacteriophage T4. Nature 227, 680–685. 5432063 10.1038/227680a0

[r21] Makela, M., Galkin, S., Hatakka, A. & Lundell, T. (2002). Production of organic acids and oxalate decarboxylase in lignin-degrading white rot fungi. Enzyme Microbial Technol 30, 542–549.

[r22] Martinez, D., Larrondo, L. F., Putnam, N., Gelpke, M. D. S., Huang, K., Chapman, J., Helfenbein, K. G., Ramaiya, P., Detter, J. C. & other authors (2004). Genome sequence of the lignocellulose degrading fungus *Phanerochaete chrysosporium* strain RP78. Nat Biotechnol 22, 695–700. 15122302 10.1038/nbt967

[r23] Micales, J. A. (1997). Localization and induction of oxalate decarboxylase in the brown-rot wood decay fungus *Postia placenta*. Int Biodeterior Biodegrad 39, 125–132.

[r24] Miller, G. L. (1959). Use of dinitrosalicilic acid reagent for determination of reducing sugars. Anal Chem 31, 426–428.

[r25] Orth, A. B., Royse, D. J. & Tien, M. (1993). Ubiquity of lignin degrading peroxidases among various wood-degrading fungi. Appl Environ Microbiol 59, 4017–4023. 8285705 10.1128/aem.59.12.4017-4023.1993 PMC195861

[r26] Pease, E. A. & Tien, M. (1992). Heterogeneity and regulation of manganese peroxidases from *Phanerochaete chrysosporium*. J Bacteriol 174, 3532–3540. 1592808 10.1128/jb.174.11.3532-3540.1992 PMC206038

[r27] Tien, M. (1987). Properties of ligninase from *Phanerochaete chrysosporium* and their possible applications. Crit Rev Microbiol 15, 141–168. 3322681 10.3109/10408418709104456

[r28] Tien, M. & Kirk, T. K. (1988). Lignin peroxidase of *Phanerochaete chrysosporium*. Methods Enzymol 161, 238–249.

[r29] van Rensburg, H., Anterola, A. M., Levine, L. H., Davin, L. B. & Lewis, N. G. (2000). Monolignol compositional determinants in loblolly pine: aromatic amino acid metabolism and associated rate-limiting steps. In *Lignin: Historical, Biological, and Materials Perspectives*, pp. 118–144. Washington, DC: American Chemical Society.

[r30] Van Soest, P. J. (1994). *The Nutritional Ecology of the Ruminant*, 2nd edn. Ithaca, NY: Cornell University Press.

[r31] Vanden Wymelenberg, A., Sabat, G., Martinez, D., Rajangam, A. S., Teeri, T. T., Gaskell, J., Kersten, P. J. & Cullen, D. (2005). The *Phanerochaete chrysosporium* secretome: database predictions and initial mass spectrometry peptide identifications in cellulose-grown medium. J Biotechnol 118, 17–34. 15888348 10.1016/j.jbiotec.2005.03.010

[r32] Vanden Wymelenberg, A., Minges, P., Sabat, G., Martinez, D., Aerts, A., Salamov, A., Grigoriev, I., Shapiro, H., Putnam, N. & other authors (2006). Computational analysis of the *Phanerochaete chrysosporium* v2.0 genome database and mass spectrometry identification of peptides in ligninolytic cultures reveal complex mixtures of secreted proteins. Fungal Genet Biol 43, 343–356. 16524749 10.1016/j.fgb.2006.01.003

[r33] Wilson, L. M. & Howlett, B. J. (2005). *Leptosphaeria maculans*, a fungal pathogen of *Brassica napus*, secretes a subtilisin-like serine protease. Eur J Plant Pathol 112, 23–29.

[r34] Yano, T., Ashida, S., Tachiki, T., Kumagai, H. & Tochikura, T. (1991a). Development of a soft gel cultivation method. Agric Biol Chem 55, 379–385. 1368691

[r35] Yano, T., Ashida, S., Tachiki, T., Kumagai, H. & Tochikura, T. (1991b). Production and localization of enzymes on soft gel cultivation. Agric Biol Chem 55, 387–391. 1368691

[r36] Yoshida, M., Igarashi, K., Kawai, R., Aida, K. & Samejima, M. (2004). Differential transcription of *β*-glucosidase and cellobiose dehydrogenase genes in cellulose degradation by the basidiomycete *Phanerochaete chrysosporium*. FEMS Microbiol Lett 235, 177–182. 15158279 10.1016/j.femsle.2004.04.032

[r37] Young, H. E. & Guinn, V. P. (1966). Chemical elements in complete mature trees of 7 species in Maine. TAPPI (Tech Assoc Pulp Pap Ind) 49, 190–195.

